# Variant signaling topology at the cancer cell–T-cell interface induced by a two-component T-cell engager

**DOI:** 10.1038/s41423-020-0507-7

**Published:** 2020-07-24

**Authors:** Dina Kouhestani, Maria Geis, Saed Alsouri, Thomas G. P. Bumm, Hermann Einsele, Markus Sauer, Gernot Stuhler

**Affiliations:** 1 Department of Internal Medicine II, Hematology and Oncology, University Clinic Würzburg, Würzburg, Germany; 2 Department of Biotechnology and Biophysics, University Würzburg, Würzburg, Germany

**Keywords:** Immunotherapy, Tumour immunology

Bispecific T-cell-engaging antibodies (BiTEs) convey antigenic information from cancer to the T-lymphocyte-associated CD3 complex, thus redirecting immune effector cells to induce target cell lysis. Since antigenic information from transformed tissues is rarely unambiguous, we recently introduced novel T-cell-engaging antibody fragments called hemibodies for the combinatorial, ultraprecise targeting of cancer.^1^ Here, we report the unprecedented enhancement of the plasticity of immunological synapse (IS) formation and variant signaling topology at the cancer cell–T-cell interface induced by hemibodies compared to that induced by BiTEs.

Physiologically, T lymphocytes are activated via invariant CD3 chains after binding of the T-cell receptor (TCR) to cognate antigen: HLA complexes presented by target cells within the context of the IS. This process involves a series of intracytoplasmic signaling events in T cells, which are initiated by the removal of an inhibitory phosphate moiety from Tyr505 in the SRC family protein tyrosine kinase LCK by CD45 phosphatases.^2^ Seconds after the dephosphorylation of LCK, the CD45 molecule is excluded from the contact zone and the newly formed central supramolecular antigen cluster (SMAC) region. This is a prerequisite for the phosphorylation of immune receptor tyrosine-based activation motifs (ITAMs) in the CD3zeta chains and the subsequent recruitment and activation of ZAP70. ZAP70 activates a number of signaling molecules to promote proliferation, exocytosis of lytic granules, and de novo synthesis of cytokines^3,4^

Bispecific antibodies redirect T cells against cancer by linking tumor-associated antigens and CD3, thus mimicking the physiological T-cell activation process (Fig. 1a, left)^5,6^ Although this is highly effective in eliminating tumor cells, a number of dose-limiting effects have been observed due to the expression of the antigen of interest on nontumor tissues^7^ or nonspecific T-cell activation. To overcome these limitations, we developed combinatorial T-cell-engaging pro-drugs named hemibodies.^1^ Hemibodies are complementary antibody fragments composed of an antigen-specific single-chain variable fragment (scFv) fused to either the variable heavy (VH) or the variable light chain domain (VL) of a CD3-binding paratope (Fig. 1a, right). In the situation where both hemibodies simultaneously bind to the surface of a given target cell, the variable domains align and reconstitute the CD3-binding site to redirect effector T cells. A proof of principle was performed in an HLA-mismatched, allogeneic transplantation model in vitro and in vivo by employing HLA-A2 and CD45 target antigens to uniquely tag and eliminate diseased hematopoietic cells in a leukemia patient. This combinatorial AND gate operation has been shown to differentiate hemibody-operated immunotherapy from therapy with BiTE molecules, which served as template structures.

**Fig. 1:**
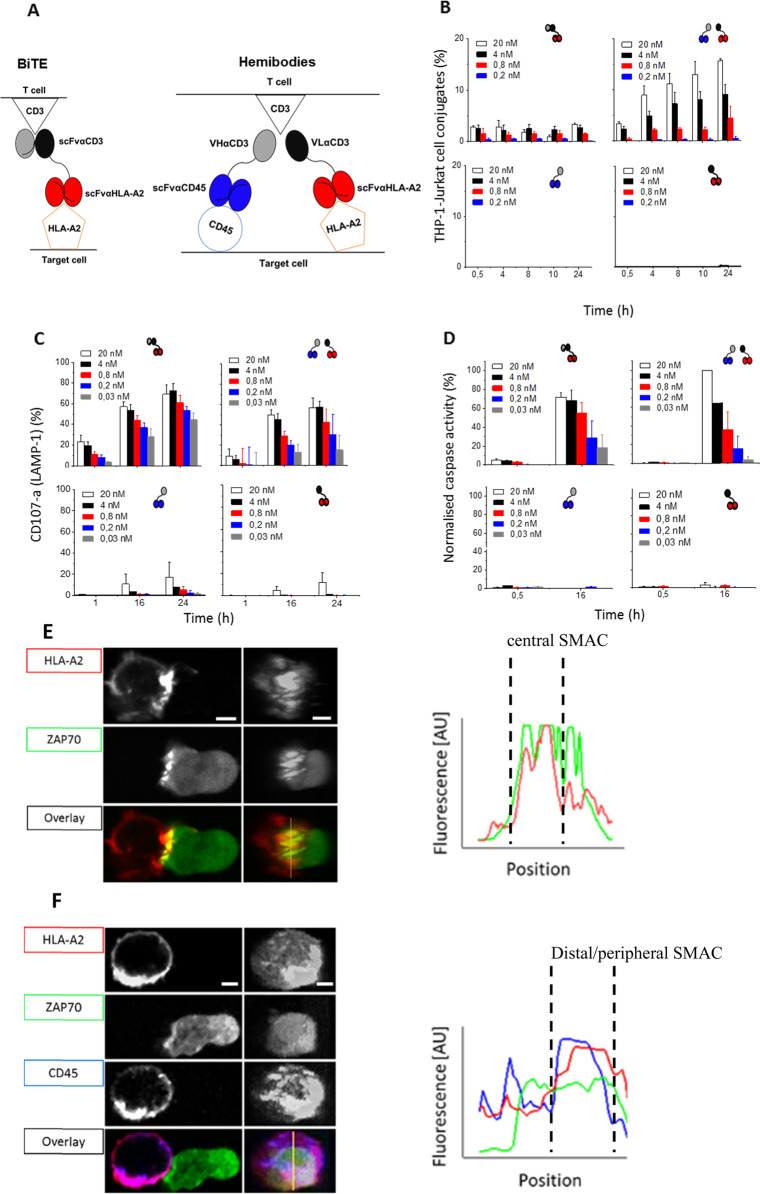
Comparable T cell engagement but variant immunological synapse formation induced by BiTEs and combinatorial hemibodies. **a** Scheme of the redirection of tumor cell targeting by a bispecific T-cell engager (BiTE; scFvαCD3-αHLA-A2) (left) or by binding of two hemibodies (VHαCD3-scFvαCD45 and VLαCD3-scFvαHLA-A2) to a combination of two antigens (HLA-A2 and CD45) and subsequent complementation of a CD3- specific paratope to engage T-cells  (right). By employing HLA-A2 and CD45 dual antigen-positive THP-1 target cells and CD3-positive Jurkat T cells, the comparable T-cell engagement potency of BiTEs and hemibodies was found, as assessed by T-cell–tumor cell conjugate formation (**b**), expulsion of lytic granules from the T-cell side (**c**) and caspase induction in tumor cells (**d**). For reconstruction of the immunological synapse (IS) and visualization of ZAP70 translocation, CD45 and HLA-A2 antigens on THP-1 target cells were stained with fluorescently labeled BiTEs or hemibodies as indicated and coincubated with Jurkat cells transfected with fluorescent ZAP 70 (**e**, **f**). Representative confocal views of THP-1 target (left) and Jurkat T cells (right) and en face views of the IS are presented. +Line profiles in the graph indicate the intensities of HLA-A2, CD45 and ZAP70 labeling along the yellow line in the contact zone

In the first set of experiments, we compared conjugate formation between CD45- and HLA-A2-positive THP-1 leukemia cells and the CD3-positive Jurkat T-cell line mediated by BiTEs and hemibodies. Conjugate formation is initiated by adhesion molecules and enhanced by TCR-triggered signals involved in shaping the mature IS. Of note, the BiTEs were directed against HLA-A2 antigens exclusively expressed by the target cells but not the HLA-A2-negative Jurkat T-cell population. As shown in Fig. 1b, we observed that hemibodies induced the formation of a higher number of conjugates than BiTEs, but this only occurred when both complementary constructs were investigated. No target cell–T-cell clusters were observed in experiments in which individual hemibody constructs were probed, emphasizing the strict need for the combination of the components of this novel device for productive T-cell engagement to occur.

To bring these results into the broader context of T-cell activation and triggering of lytic effector functions, we tested the expression of CD107a on T cells^8^ (Fig. 1c) and caspase activity in target cells (Fig. 1d). CD107a (LAMP1) is associated with cytolytic organelles and can be detected on the surfaces of T lymphocytes as a result of hemibody- or BiTE-induced degranulation after reorganization of the synapse and the cytoskeleton by microtubule organizing center (MTOC) molecules (Fig. 1c). Cleavage of intracellular caspase 3 was visualized and quantified as a marker for apoptosis in the target cell compartment.^9^ We found that both, BiTEs and the hemibody pair, but not individual hemibodies, induced the degranulation of lytic granules, leading to target cell apoptosis in a dose-dependent manner (Fig. 1c, d). Interestingly, we could not detect significant differences in the cytolytic potency of the two approaches.

CD45 and HLA molecules are found at different positions in the context of the immunological synapse, with HLA-A2 antigens gathering in the central region and CD45 localizing to the distal SMAC. To better understand the mechanism of hemibody complementation to successfully activate T cells, we resorted to confocal microscopy using fluorescently labeled HLA-A2- and CD45-specific constructs. To investigate and visualize the site of T-cell engagement, the CD3-positive Jurkat cell line was transduced with fluorescently tagged ZAP70. For the HLA-A2-specific BiTEs, we detected the formation of a typical bull’s eye-shaped immunological synapse^10^ with BiTEs bridging CD3 and HLA-A2 molecules, which were organized in microclusters, on the central target cell–T-cell interface to recruit ZAP70 to the central SMAC (Fig. 1e). For the hemibody pair interacting with the CD45 and HLA-A2 antigens, we detected both constructs in the periphery of the synapse, where they formed a ring-shaped structure by excluding HLA-A2 from the central region and translocating the molecules into the peripheral/distal region of the supramolecular activation complex. Here, ZAP70 associated in opposition to the CD45 and HLA-A2 molecules in the peripheral/distal SMAC (Fig. 1f).

Taken together, the results demonstrated that HLA molecules, when interacting with BiTEs, gather in the central SMAC of the immunological synapse, but they are excluded from the center in the distal/peripheral SMAC by hemibodies and colocalize with CD45, thus forming a ring-shaped variant IS. Although the efficacy of effector T-cell activation by BiTEs and hemibodies is quite comparable, we show that hemibody-induced synapses are dynamic in nature and that CD3 signaling takes place in the periphery and at the uropod structure of the T-cell-target cell interface. These findings reveal an unexpectedly high degree of plasticity in the formation process of the IS, both structurally and functionally. Moreover, the data reported here indicate that hemibodies can complement each other to evoke T-cell effector functions even in situations where the target antigens are in opposite positions within the topographic organization of the IS.

## Supplementary information

Supplemental
