# Supplementary material for: Variant signaling topology at the cancer cell–T-cell interface induced by a two-component T-cell engager
Source: Cell Mol Immunol. 2020 Jul 24;18(6):1568–70. doi: 10.1038/s41423-020-0507-7 (PMC8166904; doi:10.1038/s41423-020-0507-7)
Supplement: Supplementary file 1 — Supplemental [file 41423_2020_507_MOESM1_ESM.docx]

**Supplement**

**Materials and Methods**

**Construction of BiTEs and hemibodies**

The DNA coding for the HLA-A2 specific BiTE for production in E. coli was generated using the scFv sequence derived from a human antibody specific for HLA-A2 (clone 3PF12, kindly provided by Nicholas A. Watkins, University of Cambridge, UK) [1]. The scFv sequence addressing CD3ε was derived from diL2K, the de-immunized version of the mouse monoclonal antibody L2K, [2] and the scFv fragment targeting CD45 was derived from the murine IgG1 antibody BC8 [3]. An Fh8-tag was fused to the N-terminal end for enhancement of expression into the cytoplasm [4] and an 8 histidin-tag was fused to the C-terminus for purification.

DNA coding for Hemibodies were synthesized by Thermo Scientific and expressed in CHO cells. An N-glycolisation site was inserted into the GS linker between VH and VL of the antigen specific scFv domain to enhance production. In addition, a signal peptide for secretion of the recombinant protein out of the cell, as well as a Kozak consensus sequence and a NgoMIV cleavage site was fused at the 5'-end of the construct. A Twin-strep-tag II was added to the 3'-end for purification of the product. To optimize translation, codon optimization for Cricetulus griseus was performed. The designed DNA constructs were synthesized by BioCat GmbH.

**Purification of BiTEs and hemibodies**

E.coli cells were disrupted at 2-8 °C using a French Press. Proteins were loaded onto a 5 ml HiTrapTALON crude column (GE Healthcare©) connected to the ÄKTA start chromatography system (GE Healthcare Bio-Sciences, PA, USA) and washed with 5 column volumes (CV) IMAC wash buffer (50 mM Na-phosphate pH 7.5, 300 mM NaCl, 10 mM Imidazole pH 8.0), 50 CV IMAC endotoxin removal buffer (50mM Na-phosphate pH 7.5, 300 mM NaCl, 5 mM Imidazole pH 8.0, 0.2% Triton X-114), followed by 10 CV IMAC wash buffer at 5 ml/min to remove impurities and endotoxins. Finally, the His-tagged protein was eluted with 5 CV IMAC elution buffer (50 mM Na-phosphate pH 7.5, 300 mM NaCl, 150 mM Imidazole pH 8.0). Further purification of the eluates was performed on a MonoQ anion exchange chromatography column (AIEX) at 2-8°C using AIEX binding buffer (50 mM Na-phosphate pH 7.5, 75 mM NaCl) and a HiPrep 26/10 desalting column (GE Healthcare©) and a 1 ml HiTrap Q FF column (GE Healthcare©). Eluted proteins were polished by size exclusion chromatography (SEC) on a HiLoad 16/600 Superdex 200 pg column (GE Healthcare©) using a 50 mM Na-phosphate pH 7.5, 300 mM NaCl buffer as an eluent at a flow rate of 1 ml/min.

For purification of CHO derived twin-strep-tagged hemibodies, the supernatants were loaded on an equilibrated Strep-Tactin column (Econo-Column® Bio-Rad) using Strep-Tactin®XT technology according to the manufacturer’s instructions (iba life science). To isolate monomeric proteins, Strep-Tactin eluates were loaded on a HiLoad 16/600 Superdex 200 pg column and eluted with ATS buffer (200 mM L-Arginine + 20 mM Tris + 10 mM Succinic acid; pH 7.4 – 8) at 4 °C. Hemibodies and BiTEs were stored at a concentration of ~1mg/ml in PBS at 4°C in the absence of any stabilizers.

**Cell lines**

PBMCs were isolated from peripheral blood of healthy donors in agreement with institutional guidelines by density-gradient centrifugation. T cells were obtained from HLA-A2 negative donors and isolated using the Human CD3+ T cell isolation Kit (Milteny Biotech). The human cell line THP-1 (acute myeloid leukemia, ATCC TIB-202 and DSMZ ACC-16) was used as target cells and the CD3 postive Jurkat cell line (T-cell leukemia, DSMZ ACC-282) as a T cell surrogate. All cells were grown in advanced RPMI-1640 supplemented with 200 μM L-glutamine, 10% FBS, penicillin (200 U/mL) and streptomycin (200 μg/mL) (Thermo Fisher Scientific).

**Fluorescent Labeling of Hemibodies and BiTEs**

Proteins were labeled at 1 mg/ml in PBS with Alexa fluor 647, 546, and 532 using the appropriate protein labeling kit (Thermo Fisher Scientific).

**Confocal microscopy and imaging analysis**

To analyze the IS formation and ZAP70 activation mediated by hemibodies and BiTEs, 25.000 THP-1 cells were incubated with the respective constructs at 3 μg/ml in 100 μl PBS for 30 min on ice in the dark. To remove unbound proteins, the cells were washed one time with cold PBS and stored on ice until microscopy. Shortly before imaging, cells were warmed up, co-incubated with 25.000 Jurkat or ZAP70-GFP transduced Jurkat cells in 200μl PBS and transferred onto microscopy slides (15 μ-slide 8 well Glass Bottom ibidi). Images were acquired using an inverted IX81 microscope equipped with an Olympus FV1000 confocal laser scanning system, a FVD10 SPD spectral detector and diode lasers of 405, 473, 559, and 635 nm. Representative images were acquired using Olympus UPLSAPO60x (oil, numerical aperture: 1.35) and/or Olympus UPLFLN 40x (oil, numerical aperture: 1.30) objectives. For high-resolution confocal scanning, an automatic pinhole setting was employed. The resolution was set with at least 2 pixels per micron in x-y direction. Z-stack images were processed by maximum intensity projection and the noise was reduced using despeckle function and then cropped to focus on the cell of interest. Images are presented as RGB (32 bits per pixel) and processing was performed using Image J software.

**Functional assays**

For the conjugation assay, 50.000 THP-1 target cells were labeled with CellTracker™ Deep Red Dye and mixed with 250.000 CellTracker™ Green CMFDA Dye labelled Jurkat T cells (target cell: T cell ratio= 1:5) in 96-well plates (Costar®, Corning Inc., USA). Different concentrations of constructs were added as indicated and incubated using standard cell culture conditions (37°C, 5% CO2) (The incubation periods varied in different experiments as indicated).

The appearance of the lysosomal marker CD107a (LAMP-1) was utilized as a measurement for cytolytic activity. For the detection of CD107a (LAMP-1) positive T cells, 1 x 105 THP-1 target cells were mixed with 5 × 105 CD3+ PBMCs from a HLA-A2 negative healthy donor (target cell: T cell ratio= 1:5) and stained with α-CD107-a (LAMP-1)-APC antibody (BioLegend). Cells were gated for CD8 and CD107a positivity using flow cytometry techniques (FACS).

T cell stimulation and concomitant CD3 down regulation was induced by BiTEs and hemibodies as described. After 16 h, the decrease of CD3 molecules per cell was assessed using the PerCP/Cyanine5.5 anti-human CD3 Antibody (BioLegend) and quantified by FACS.

To detect intracellular caspase activity in target cells, co-cultures of CD3+ HLA-A2- T lymphocytes and THP-1 cells were stimulated by BiTEs and hemibodies as described. After 16 h, a caspase specific fluorescent substrate (Biotium) was added and incubated for 30 min at RT, followed by measuring the fluorescent signal by FACS.

1. Watkins, N.A., et al., *The isolation and characterisation of human monoclonal HLA-A2 antibodies from an immune V gene phage display library.* Tissue Antigens, 2000. **55**(3): p. 219-28.
2. Brischwein, K., et al., *MT110: a novel bispecific single-chain antibody construct with high efficacy in eradicating established tumors.* Mol Immunol, 2006. **43**(8): p. 1129-43.
3. Lin, Y., et al., *A genetically engineered anti-CD45 single-chain antibody-streptavidin fusion protein for pretargeted radioimmunotherapy of hematologic malignancies.* Cancer Res, 2006. **66**(7): p. 3884-92.
4. Costa, S.J., et al., *The novel Fh8 and H fusion partners for soluble protein expression in Escherichia coli: a comparison with the traditional gene fusion technology.* Appl Microbiol Biotechnol, 2013. **97**(15): p. 6779-91.
